# What’s in a Name? Effect of Breed Perceptions & Labeling on Attractiveness, Adoptions & Length of Stay for Pit-Bull-Type Dogs

**DOI:** 10.1371/journal.pone.0146857

**Published:** 2016-03-23

**Authors:** Lisa M. Gunter, Rebecca T. Barber, Clive D. L. Wynne

**Affiliations:** 1 Department of Psychology, Arizona State University, Tempe, Arizona, United States of America; 2 Mary Lou Fulton Teachers College, Arizona State University, Tempe, Arizona, United States of America; Oregon State University, UNITED STATES

## Abstract

Previous research has indicated that certain breeds of dogs stay longer in shelters than others. However, exactly how breed perception and identification influences potential adopters' decisions remains unclear. Current dog breed identification practices in animal shelters are often based upon information supplied by the relinquishing owner, or staff determination based on the dog's phenotype. However, discrepancies have been found between breed identification as typically assessed by welfare agencies and the outcome of DNA analysis. In Study 1, the perceived behavioral and adoptability characteristics of a pit-bull-type dog were compared with those of a Labrador Retriever and Border Collie. How the addition of a human handler influenced those perceptions was also assessed. In Study 2, lengths of stay and perceived attractiveness of dogs that were labeled as pit bull breeds were compared to dogs that were phenotypically similar but were labeled as another breed at an animal shelter. The latter dogs were called "lookalikes." In Study 3, we compared perceived attractiveness in video recordings of pit-bull-type dogs and lookalikes with and without breed labels. Lastly, data from an animal shelter that ceased applying breed labeling on kennels were analyzed, and lengths of stay and outcomes for all dog breeds, including pit bulls, before and after the change in labeling practice were compared. In total, these findings suggest that breed labeling influences potential adopters' perceptions and decision-making. Given the inherent complexity of breed assignment based on morphology coupled with negative breed perceptions, removing breed labels is a relatively low-cost strategy that will likely improve outcomes for dogs in animal shelters.

## Introduction

Morphological differentiation in the domestic dog is evident in the diversity of modern breeds from the Chihuahua to the Irish Wolfhound [1]. Along with a range of body shapes and sizes, dog breeds have also undergone selection for behavioral traits to aid working needs such as hunting, herding, and protection [2]. Accompanying this human influence, both companion animal professionals [3] as well as members of the public who are less familiar with dog breeds [4,5], have developed beliefs about breed-typical behavior.

Around 83.3 million dogs live in human households in the United States with approximately 20% of these dogs having been adopted from animal shelters [6]. In a 1999 survey of 186 shelters across the country, dogs stayed an average of 9.5 days in the shelter, where over half of them were euthanized, and the others were either adopted or reclaimed by their owners [7]. It is currently estimated that approximately 3.9 million dogs enter animal shelters each year and over 30% are euthanized [8].

Many individual qualities of dogs have been found to correlate with adoption success, especially morphology. Weiss, Miller, Mohan-Gibbons, and Vela [9] found appearance to be the single most important reason adopters gave for choosing their new dog; and in Protopopova, Gilmour, Weiss, Shen, and Wynne [10], potential adopters were able to distinguish between dogs that had been adopted or euthanized based solely on their attractiveness in photographs. Ramirez [11] reported that dog owners recalled appearance, personality and attraction to their dog as the reasons for choosing their current dog; and Nemcova and Novak [12] found that over one-third of respondents rated appearance as the most important factor in dog selection. Given that physical appearance is important to those looking to adopt dogs, we are interested in how breed labels influence that attractiveness.

Conventionally in the United States, the term “pit bull” has been applied to breeds such as American and English bulldogs, Staffordshire bull terriers, American Staffordshire terriers and American Pit Bull terriers, as well as mixes of these and other breeds [13]. Earlier studies of dogs in shelters by Posage, Bartlett, and Thomas [14] and Lepper, Kass, and Hart [15] found that pit bulls, as well as wolf hybrids and feral animals, were typically not made available to the public for adoption and were euthanized based on shelter policies. More recently in Protopopova et al. [10], American Pit Bull terriers were found to be the most prevalent breed available for adoption; and in Dowling-Guyer, Marder, and D’Arpino [16], Pit Bull terriers were the most common breed evaluated at the shelter. Studies examining adoption success in US shelters [17,10,18,19] have found breed designation to be associated with differing outcome rates, such as increased euthanasia and length of stay. Exactly how breed identification influences potential adopters’ decisions to take a dog home with them remains unclear.

Negative perceptions of certain breeds of dogs, particularly about pit-bull-type breeds, may be influenced by reports of aggression towards humans, including incidents of dog bite injuries and deaths [20–24]. With the Pit Bull Terrier’s bullbaiting and dogfighting history, this breed often demonstrates an increased propensity for aggression towards other dogs and other animals, with an intensity of destructiveness in its attacks, which likely contributes to such perceptions [13,25]. While an association may exist between certain types of dogs and human-directed aggression, the reliability of breed characterization in positively identifying dogs involved in these types of incidents is controversial and debated [13,26].

In animal shelters, dog breed identification practices are often based upon owner reports or staff determination according to the dog’s appearance. Research by Voith, Ingram, Mitsouras, and Irizarry [27] and Voith et al. [28] has found that discrepancies exist between breed identification by animal shelters and DNA analysis. Specifically, breed identification of pit-bull-type dogs by shelter staff and veterinarians was shown to be inconsistent among individuals and an unreliable means of identification. In particular, 50% of dogs labeled as pit bulls lacked DNA breed signatures of breeds commonly classified as pit bulls [29].

The aim of the studies reported here is to build on previous work investigating handler appearance and breed perceptions [5,30] by examining the effects of breed labeling on perceived attractiveness and outcomes in the animal shelter setting. Prior research has indicated that a majority of dogs arriving into shelters are of mixed breeds [31,32]. Given that some breeds of dogs are adopted less and stay longer in shelters than others, and breed identification based on visual identification is unreliable, we want to know how perceptions of breeds and differences in labeling can impact outcomes for shelter dogs.

Study 1 investigated perceived attractiveness of three dog breeds (Labrador Retriever, pit bull and Border Collie) and the influence of different human handlers on that dog’s attractiveness when viewed in photographs. In Study 2, we compared length of stay and potential adopters’ perceptions of unlabeled photographs of pit-bull-type dogs and dogs that looked indistinguishable from those dogs but had been assigned to a different breed (which we are calling here “lookalikes”) by staff at an animal shelter. In Study 3, potential adopters viewed pit-bull-type dogs and lookalikes in videos with and without breed labels to assess the effect of these labels on perceived attractiveness. For Study 4, data was collected from an open admission animal shelter in Florida before and after breed assignment was no longer made available to the public on kennel cards and online adoption profiles. We analyzed lengths of stay and outcomes for all breed groups, including pit bulls.

## Study 1

The objective of this study was to investigate the impact of breed impressions and the effects of handler appearance on the perceptions of pit bulls. Our prediction was that the pit-bull-type dog would have lower behavioral desirability and adoptability ratings compared to the other dogs, and that the appearance of a male child and elderly woman alongside the pit bull would improve perceptions of the dog, while a rough adult male would negatively impact them.

### Method

#### Participants

The study involved 49 participants from psychology classes at Foothill College (Los Altos, CA, USA) who received credit in exchange for their participation, and 179 members of the online community forum, Reddit, who participated without compensation for their involvement.

#### Procedure

All procedures were approved by the Foothill De Anza Community College Institutional Research and Planning Office. Upon agreeing to complete a survey about human-animal relationships, Foothill College participants were given access to a campus computer where they completed the informed consent form and online survey. Participants from the website Reddit were made aware of the study through a survey request posting, and completed the informed consent form and survey online. The survey was offered through the research software company Qualtrics (Provo, UT, USA).

The survey included eight dog experience questions about ownership, source of dog, breeds owned, dog bite history, professional and volunteer experience with dogs, and three demographic questions concerning age, gender and race of the participant. The survey showed a randomized sequence of three images without breed labels: a Labrador Retriever, a pit-bull-type dog and a Border Collie. Dogs in these images were in a sitting position, all occupying approximately the same amount of space in the image, and no humans or other animals were visible in the picture. In the second randomized sequence each dog was shown with a human handler. The Labrador Retriever was shown with a middle-aged woman in a wheelchair, the Border Collie with a middle-aged athletic male and either a tattooed adult male, elderly woman or male child was shown with the pit bull. In these dog/handler images, the dogs were either in a sitting or standing position next to a handler with both dogs and handlers occupying approximately the same amount of space in the image. All images were taken by a professional photographer in May 2012 (see S1 Appendix for images). Those individuals included in the images have given their written informed consent (as outlined in PLOS consent form) to publish these details.

Each survey page displayed one image with six questions about the behavior and adoptability on which the dog was rated. The six questions were: “I would feel comfortable approaching this dog,” “This dog looks smart,” “This looks like an aggressive dog,” “This dog looks friendly to me,” “This dog looks difficult to train,” and “If circumstances allowed, I’d consider adopting this dog.” Participants were asked to rate their level of agreement on a six-point Likert scale ranging from “Strongly Disagree” to “Strongly Agree” with no neutral choice. Once an image was rated on the six questions, participants were not able to return to previously completed pages.

#### Statistical Analyses

Differences in participants’ responses to behavioral and adoptability questions for the Labrador Retriever, pit-bull-type dog and Border Collie were analyzed using a one-factor repeated measures ANOVA with dependent pairwise comparisons between each of the dogs. Using the pit bull without a handler as a baseline measure of the dog’s perceived approachability, intelligence, friendliness, aggressiveness, difficulty to train and adoptability, differences in the perceptions of the rough adult male, elderly woman and male child conditions were analyzed using one-factor ANOVAs with dependent pairwise comparisons between baseline and handler.

### Results and Discussion

**Fig 1:**
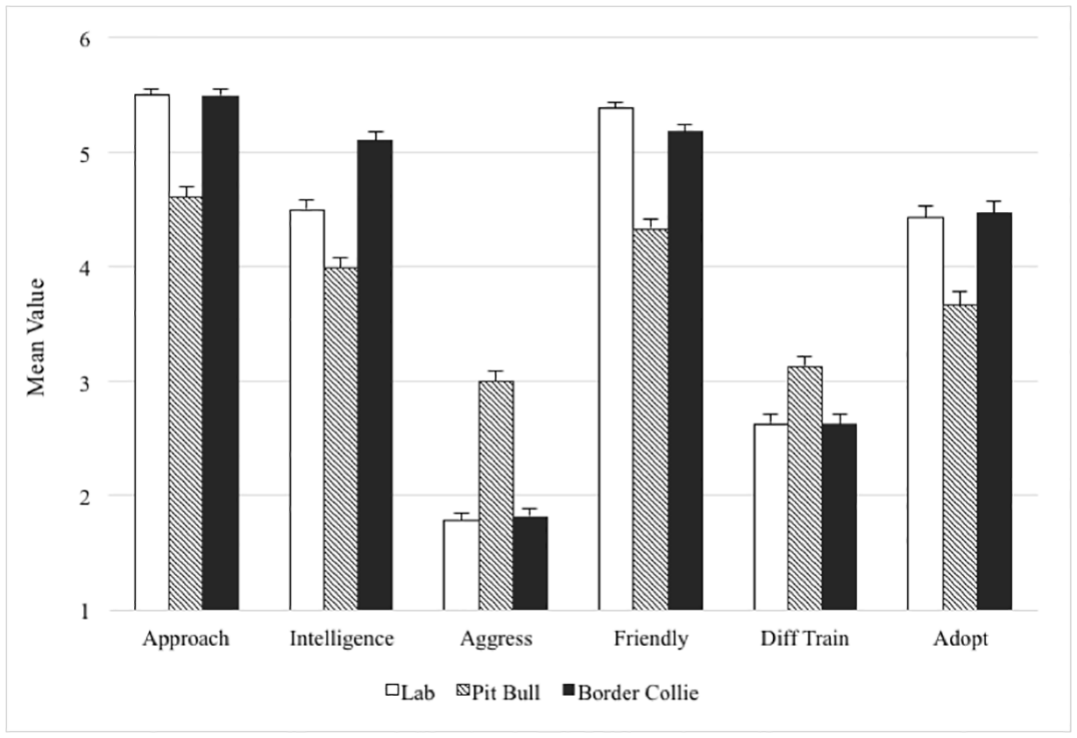
Columns represent mean values (with error bars for standard errors) on the 6-point Likert scale for each of the perceived behavioral and adoptability characteristics in response to the breed exemplar photographs of the Labrador Retriever, pit-bull-type dog and Border Collie. Paired samples t-tests indicated that all comparisons between the pit bull and other breeds were statistically significant.

One-factor repeated measures ANOVAs were used to determine the effect of breed on the dogs’ behavioral and adoptability ratings. As seen in Table 1, analysis indicated significant differences in the means of the pit-bull-type dog, Labrador Retriever and Border Collie in all six characteristics. The magnitude of differences in perceived approachability, intelligence, aggressiveness, and friendliness were consistent with large effect sizes, while adoptability and difficulty to train were medium and small, respectively [33]. Paired samples t-tests indicated that trait comparisons between the pit bull and other breeds were statistically significant (Smallest *t* = 5.42, largest = 14.27, df = 225–227, each comparison at *p* < .001) as shown in Fig 1.

**Table 1:** Behavioral & Adoptability in Breed Exemplar Photographs.

	Lab		PB		BC				
Characteristic	*M*	*SE*	*M*	*SE*	*M*	*SE*	*F*	*p*	*η2*
Approachability	5.50	0.05	4.61	0.08	5.49	0.05	113.54	<.001	0.17
Intelligence	4.50	0.07	3.99	0.08	5.10	0.07	80.83	<.001	0.14
Aggressiveness	1.78	0.06	3.00	0.08	1.82	0.06	157.00	<.001	0.24
Friendliness	5.38	0.05	4.33	0.08	5.18	0.05	127.19	<.001	0.20
Difficulty to Train	2.62	0.07	3.12	0.08	2.62	0.08	19.52	<.001	0.04
Adoptability	4.44	0.09	3.67	0.10	4.47	0.09	40.84	<.001	0.06

*Note*: Characteristic mean values and standard errors for the Labrador Retriever, pit-bull-type dog and Border Collie. One-factor repeated measures ANOVAs were performed on each characteristic comparing the three dogs with corresponding F and p values. η^2^ is an effect size measure indicating the magnitude of the difference between the three dogs.

**Fig 2:**
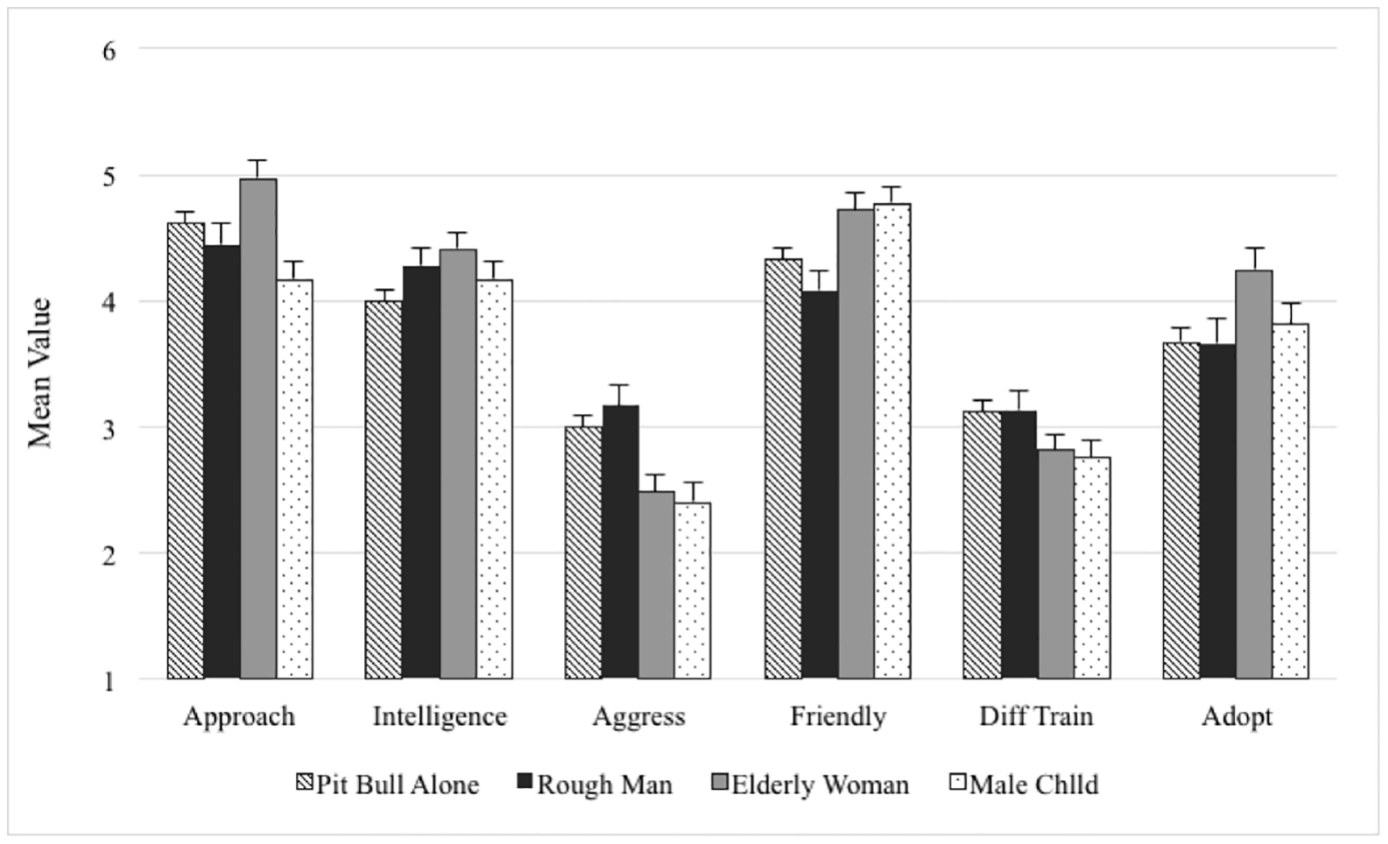
Columns represent mean values (with error bars for standard errors) on the 6-point Likert scale for each of the behavioral and adoptability characteristics for the pit bull when presented alone, with a rough adult male, elderly woman and male child. Perceived intelligence improved in all handler conditions. Approachability, friendliness, and adoptability improved and aggressiveness was reduced with the elderly woman. Friendliness and adoptability improved while aggression and difficulty to train lessened with the male child. With the rough male, perceived friendliness decreased.

One-factor ANOVAs were utilized to assess the influence of handlers on the pit bull’s perceived behavioral traits and adoptability. As described in Table 2, the presence of a handler alongside the pit bull significantly changed baseline measures (i.e., when viewed alone) of the dog in all six traits. As shown in Fig 2, paired samples t-tests indicated that perceived intelligence increased with all handlers. Approachability, friendliness and adoptability increased while aggressiveness decreased with the presence of the elderly woman. The male child improved perceptions of friendliness and adoptability of the pit bull while lessening the pit bull’s aggressiveness and perceived difficulty to train. The rough adult male reduced perceived friendliness of the pit bull.

**Table 2:** Behavioral & Adoptability of Pit Bull Viewed Without & With Handlers.

	PB		RM		EW		MC			
Characteristic	*M*	*SE*	*M*	*SE*	*M*	*SE*	*M*	*SE*	*F*	*p*
Approachability	4.61	0.08	4.45	0.16	4.97*	0.14	4.76*	0.15	2.55	0.055
Intelligence	4.61	0.08	4.27*	0.13	4.41*	0.97	4.17*	0.13	3.33	0.019
Aggressiveness	3.00	0.08	3.17*	0.15	2.48*	0.13	2.40*	0.15	8.10	< .001
Friendliness	4.35	0.08	4.08	0.15	4.72*	0.12	4.77*	0.12	6.58	< .001
Difficulty to Train	3.16	0.08	3.13	0.14	2.81	0.11	2.75*	0.13	3.60	0.014
Adoptability	3.67	0.10	3.65	0.20	4.24*	0.16	3.81*	0.16	2.81	0.039

*Note*: Characteristic mean values and standard errors for the pit bull as seen alone, with the rough male (RM), elderly woman (EW) and male child (MC). One-factor ANOVAs were performed on each characteristic comparing the four conditions with corresponding F and p values. Asterisks next to mean handler values indicate significant differences compared to the pit bull when viewed alone as indicated by paired sample t-tests.

With the lowest ratings in perceived approachability, intelligence, friendliness and adoptability and the highest in aggressiveness and difficulty to train, these results indicate that participants perceived the pit bull as the least attractive among the dogs presented. The elderly woman and male child had a positive multi-dimensional impact, while the addition of the rough adult male demonstrated only a small negative effect on participants’ perceptions of the pit bull.

## Study 2

Study 1 showed that pit-bull-type dogs were perceived more negatively than the other breeds when students and Reddit participants viewed studio photographs of breed-exemplar dogs. To our knowledge this is the first experimental demonstration of this phenomenon and we therefore decided to test whether these perceptions are also relevant in the shelter environment, where potential adopter’s preference for certain breed types could have life or death consequences for the dogs. Study 2 tests the influence of breed labeling on length of stay in a shelter and potential adopters’ perceptions of the attractiveness of these dogs. In this study, we used photographs of mixed breed shelter dogs and recruited participants visiting a shelter for the purpose of adopting a dog. We predicted that pit-bull-type dogs would have longer lengths of stay in the shelter than dogs that look phenotypically similar but had been labeled by shelter staff as another breed (“lookalikes”). Furthermore, we predicted that when viewed in photographs without breed labels, participants would not rate the pit-bull-type dogs or lookalikes differently in attractiveness.

### Method

#### Participants

Thirty-nine potential adopters who entered the Arizona Animal Welfare League and SPCA (AAWL, Phoenix, AZ, USA) interested in adopting a dog in January and February 2014 participated in the study. AAWL is a limited-admission private animal shelter with adoptable dogs obtained from owner surrenders and municipal animal control facilities. Upon agreeing to complete a survey regarding their attitudes about dogs in animal shelters, participants were given access to a laptop computer on which they completed an informed consent form and online survey.

#### Procedure

Photographs of pit-bull-type dogs and lookalikes adopted between October 2011 and January 2014 from AAWL were collected via an online shelter inventory database, PetPoint (Oakville, ON, CAN). Lookalikes were defined as dogs that appeared in photographs to be morphologically similar in stature, head, coat color and length (as determined by the first author) but were labeled another breed by staff. Lookalikes were also matched in age (+/- 3 years) and weight (+/- 30% body weight). Upon matching 15 pairs of pit-bull-type dogs and lookalikes, length of stay data was retrieved from the database.

The survey included five dog experience questions about ownership, source of dog, dog bite history, professional and volunteer experience with dogs and three demographic questions concerning the age, gender and race of the participant. The survey showed 30 dogs (15 pit-bull-type dogs and 15 lookalikes) in a randomized sequence. All images were similar in size and quality with the dog occupying approximately the same amount of photographic space with no human present (see S2 Appendix for images).

Each survey page displayed one image with five questions about behavior and adoptability on which the dog was rated. The five questions were: “I would feel comfortable approaching this dog,” “This dog looks smart,” “This looks like an aggressive dog,” “This dog looks friendly to me,” and “If circumstances allowed, I’d consider adopting this dog.” Participants were asked to rate their level of agreement on a 6-point Likert scale ranging from “Strongly Disagree” to “Strongly Agree.” The trainability question from Study 1 was removed to improve reliability after a reliability analysis conducted on responses from Study 1, (6 items; Cronbach’s α = .81, 5 items; Cronbach’s α = .82). Once an image was rated on the five traits, participants were not able to return to previously completed pages. All procedures were approved by the Arizona State University Institutional Review Board.

#### Statistical Analyses

Length of stay data were collected from pit-bull-type dogs and lookalikes. For the purposes of our study, length of stay was defined as the number of days available for adoption. Total length of stay, from day of intake to adoption, was positively correlated with available length of stay, r (30) = .89, p < .001. For dogs that had more than one stay at the shelter, the most recent was used. Differences in mean length of stay between pit-bull-type dogs and lookalikes were analyzed using a one-factor ANOVA.

Participants’ five responses for each dog were averaged to create an attractiveness composite score. Composite scores were then normalized to correct for individual differences in the use of the rating scale [normalized composite score = (original composite score—minimum composite score) / composite range] [10]. Differences in mean attractiveness composite scores between pit-bull-type dogs and lookalikes were analyzed using a one-factor repeated measures ANOVA.

### Results and Discussion

**Fig 3:**
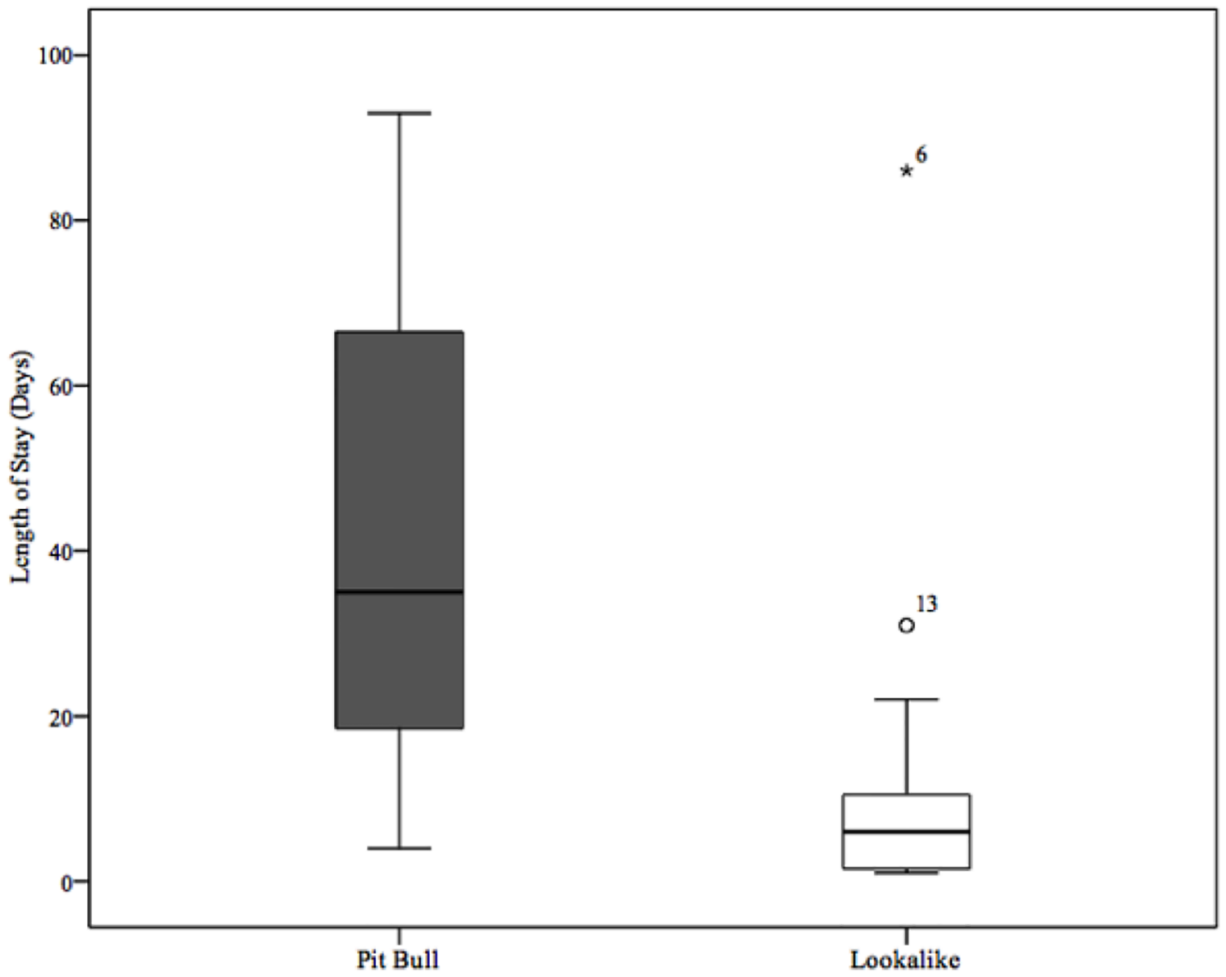
Box plots of the distribution of lengths of stay of dogs labeled as pit-bull-type and lookalike breeds. The line in the middle of the boxes represents the median (PB = 35 days, Lookalike = 6 days), the top and bottom: upper and lower 25% quartiles, respectively. Whiskers at the ends of the boxes represent the maximum values, excluding outliers.

A one-factor ANOVA was performed to assess the influence of label on length of stay. Analysis indicated significant differences between pit bull and lookalike means, *F* (1,28) = 9.29, *p* = .005. The average length of stay for pit-bull-type dogs was 42.07 days (SD = 29.98) and for lookalikes 12.80 (SD = 22.01). The *w*^2^ effect size demonstrated that the pit bull label explained 21.7% of variability in length of stay, which is a large effect size by conventional standards [33] and confirms our hypothesis of an association between assigned breed label and days available for adoption (see Fig 3 for results).

A one-factor repeated measures ANOVA was conducted to determine whether unlabeled pit-bull-type dogs and lookalikes differed in perceived attractiveness. Participants did not rate pit bulls less attractive than dogs in the lookalike group, *F* (1,14) = .007, *p* = .94. The averages of normalized composite scores for pit-bull-type dogs and lookalikes were .55 (SD = .10) and .55 (SD = .11) respectively. These values are functionally identical to the limit of our precision of measurement, indicating that, without breed labels, potential adopters in our survey did not distinguish pit-bull-type dogs from lookalikes in attractiveness.

In examining the lengths of stay of dogs labeled as pit bulls and phenotypically similar dogs labeled as another breed at AAWL, the pit-bull-type dogs averaged over three times longer stays than their lookalike matches. However when asked to rate photographs of these same dogs without breed labels, we found no difference in how attractive photographs of these two groups of dogs were seen by potential adopters. This suggests that the disparity in how long the dogs remained at the shelter waiting for adoption may have been influenced in part by the perception of the label.

## Study 3

Study 2 identified a correlation between breed labeling and length of stay in shelter dogs but detected no difference in the attractiveness of these dogs in adoption photographs. However when individuals are contemplating dog adoption, they are visiting the shelter and viewing dogs behaving in their kennels. Thus a possible limitation of Study 2 is that potential adopters making real-life choices may be influenced by aspects of a dog’s behavior that are not apparent in a still photograph. Study 3 was designed to further test the impact of breed labels on perceived attractiveness of pit-bull-type dogs and lookalikes using short videos of the dogs presented alongside manipulated breed labels in order to test the labels’ impacts on potential adopters’ perceptions of the dogs. We predicted that pit-bull-type dogs and lookalikes viewed in videos without breed labels would be rated as equally attractive. When the pit bull label was applied, we predicted it would reduce the dogs’ attractiveness ratings whereas the lookalike label would improve them.

### Method

#### Participants

Fifty-one participants who entered the AAWL interested in adopting a dog in December 2014 and January 2015 participated in the study. As in Study 2, potential adopters were recruited to complete a survey regarding their attitudes about shelter dogs; and upon agreeing to participate were provided a laptop computer on which they completed an informed consent form and online survey.

#### Procedure

Videos of pit-bull-type dogs and lookalike breeds of dogs available for adoption between May and December 2012 from Alachua County Animal Services (Gainesville, FL, USA) were used in this study. Alachua County Animal Services is an open-admission county animal shelter with dogs obtained from owner surrenders and seizures in its animal control capacity. One-minute videos using a Kodak PlaySport Zx3 video camera (Kodak Company, Rochester, NY, USA) were made of the dogs available for adoption. The videographer stood at the front of the dog’s kennel in an outside walkway area available to the public, recorded its behavior for one minute, then moved to the next kennel and repeated the procedure. These videos were originally collected and used in another study [34]. For the purposes of this study, only videos of single-housed dogs were used, and all videos were edited to fifteen seconds with the sound removed. Dogs labeled as pit-bull-type dogs and lookalike dogs were matched on morphological features and age (+/- 3 years) as in Study 2. Additionally, the dogs were matched on their in-kennel behaviors, such that both the pit-bull-type dog and its lookalike displayed similar body position, face orientation and locomotion during the fifteen seconds used in the survey.

The previous dog experience and demographic questions from Studies 1 and 2 were included here. The survey showed 10 videos of dogs (5 pit-bull-type dogs and 5 lookalikes) in a randomized sequence and then the same 10 videos with a randomized breed label. In the first display of videos, all dogs were accompanied on the screen by the legend “Available for Adoption;” in the second display, the videos included a breed label instead. Participants viewed each video once in the second set and either received the breed label used by the shelter or that of the dog’s matched lookalike. All videos were similar in size and quality with the dog occupying approximately the same amount of space on the screen with no humans present.

Each survey page displayed one video at a time with the same five behavior and adoptability questions and 6-point Likert scale used in Study 2. Once an image was rated on the five traits, participants were not able to return to previously completed pages.

#### Statistical Analyses

Participants’ five responses to the behavioral and adoptability questions for each video were averaged to create an attractiveness composite score for the dog shown. With these scores, interquartile ranges (IQR) for each participant were calculated to measure scale engagement with individual ranges between 0 and 1. Those with IQRs < 0.2 were excluded from further analysis [10]. Differences in mean attractiveness scores between pit-bull-type dogs and lookalikes without breed labels were analyzed using a one-factor repeated measures ANOVA, and attractiveness of dogs with the pit bull and lookalike breed labels was analyzed with a one-factor ANOVA. Comparisons of mean attractiveness scores analyzing changes in perceptions of the same dogs without labels and with either the lookalike or pit bull label used one-factor repeated measures ANOVAs.

### Results and Discussion

**Fig 4:**
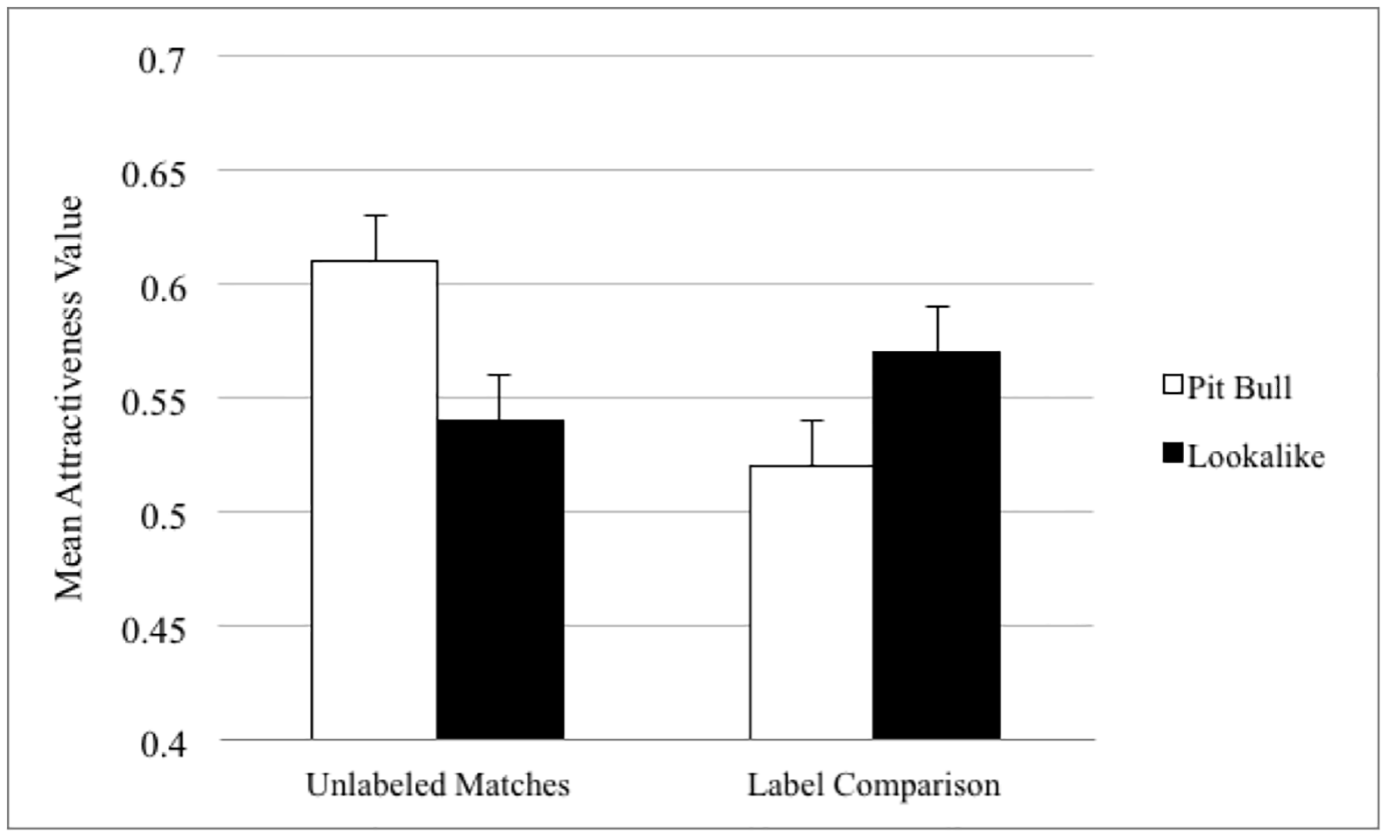
Columns represent normalized mean attractiveness values (with error bars for standard errors) for matched pit-bull-type dogs and lookalikes without labels (on left) and when dogs received either the pit bull or lookalike breed labels (on right). Without labels, pit-bull-type dogs were viewed as more attractiveness than lookalikes. With labels, participants preferred dogs with lookalike breeds.

A one-factor repeated measures ANOVA was conducted to determine whether pit-bull-type and lookalike dogs differed in perceived attractiveness when viewed without breed labels. When no breed labels were included, pit bulls were seen as more attractive than the lookalikes, *F* (1,253) = 7.42, *p* = .007, with composite score averages for pit-bull-type dogs of .61 (SD = .30) and lookalikes .54 (SD = .33). When examining the participants’ perceptions of lookalikes and pit bulls with labels, a one-factor ANOVA found that dogs with lookalike breed labels were viewed as more attractive, *F* (1,493) = 4.45, *p* = .035. Averages of attractiveness scores were .57 (SD = .29) and .51 (SD = .30), respectively (see Fig 4 for results).

**Fig 5:**
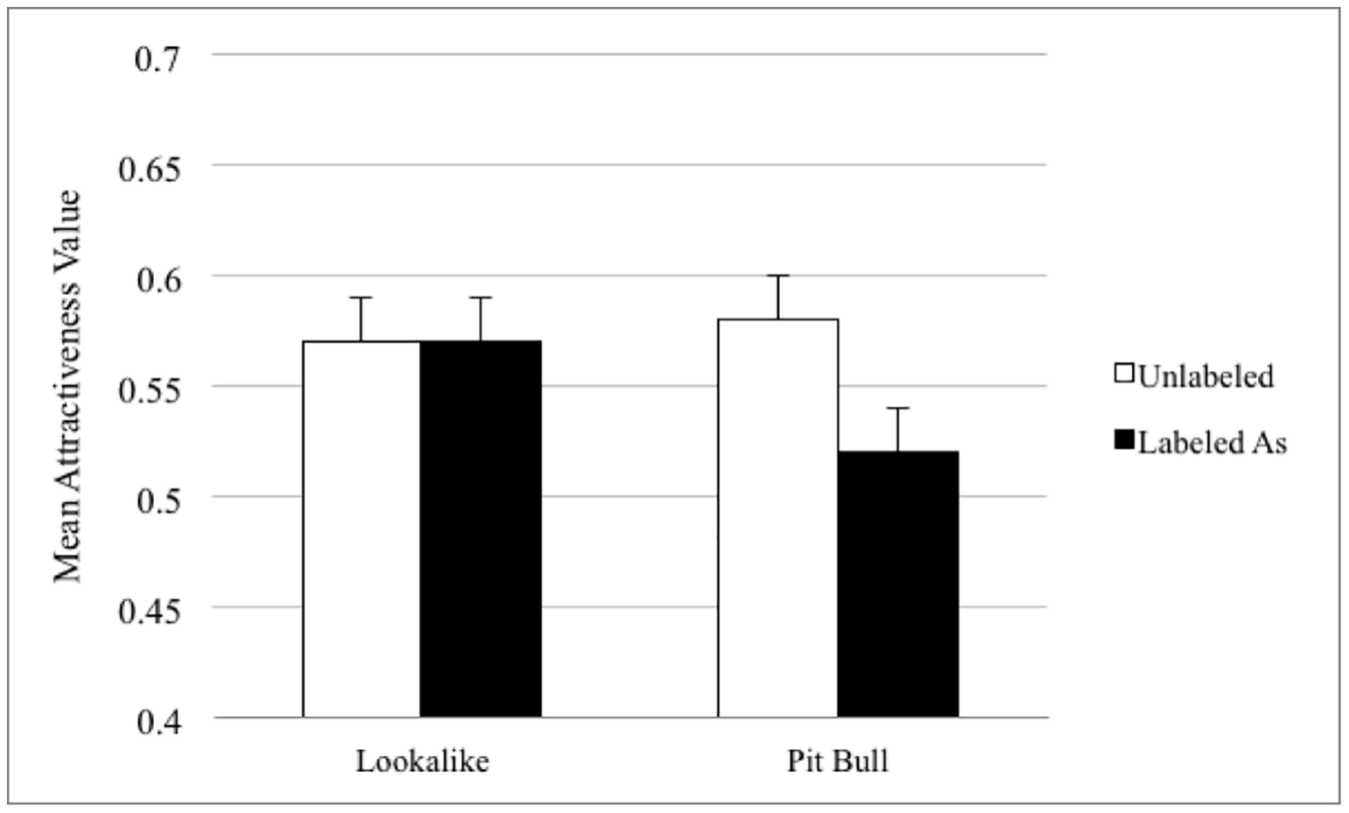
Columns represent normalized mean attractiveness values (with error bars for standard errors). On the left, dogs were rated without and with the lookalike breed labels. On the right, dogs were rated without and with the pit-bull-type breed labels. While the lookalike breeds did not change potential adopter perceptions when compared to viewing them without, dogs were perceived as less attractive when the pit bull label was used.

A one-factor repeated measures ANOVA was utilized to examine changes in perceived attractiveness of the dogs when no label was presented versus presentation with a lookalike breed label. As seen in Fig 5, participants did not rate the groups differently, *F* (1,245) = .05, *p* = .82 with average normalized composites scores without labels of: .57 (SD = .32) and with labels: .57 (SD = .29). A one-factor repeated measures ANOVA compared changes in perceived attractiveness without breed and with pit-bull-type breed labels. Dogs labeled as pit bulls were seen as less attractive than the same dogs without breed labels, *F* (1,246) = 10.68, *p* = .001. The average composite score with the pit bull label was .51 (SD = .30) and without was .58 (SD = .32).

Without seeing breed labels in these matched videos, participants viewed the dogs that had been labeled as pit bulls at the shelter as more attractive than their lookalike counterparts; however, when labels were present, potential adopters preferred dogs with lookalike labels over those with the pit bull label. To further understand the influence of the lookalike label, we compared ratings given to the same dogs when no breed label was presented and then with a lookalike breed label. We found no difference. Nevertheless when the same comparison was made with pit-bull-type breeds, participants rated the same dogs that were first seen without a label as less attractive when they were presented with the label “pit bull.” The results of these manipulations suggest that pit bull breed labels may have the ability to negatively influence perceptions of potential adopters during decision-making processes.

## Study 4

Thus far in our examinations of the influence of different labels on potential adopters’ ratings of photographs and videos of dogs, as well as shelter records of lengths of stay, we have seen that the label “pit bull” was correlated with more days awaiting adoption and poorer adopter perception; whereas when the same dogs were viewed in photographs without labels they were perceived as indistinguishable from lookalikes, and in videos pit bulls were actually preferred to lookalikes. Furthermore, the breed label attached to lookalike dogs had no detectable impact on potential adopters’ perceptions of those dogs when compared to perceptions of those dogs without labels.

Consequently, we predicted that the removal of breed labels from the information provided to potential adopters at a shelter would increase adoption of pit-bull-type dogs without impacting adoption of other breeds of dog. On February 6, 2014, Orange County Animal Services (OCAS, Orlando, FL, USA) removed breed assignments from their kennel cards and online adoption profiles in an effort to “boost adoption numbers for shelter pets” (http://www.ocnetpets.com). OCAS administration made their animal records available to us for analysis. Comparing the previous 12-month period, we predicted that in the absence of breed labels, pit bulls would have reduced lengths of stay and increased adoptions after February 6, 2014, whereas other breeds would show no changes in these outcome measures.

### Method

#### Description of Data Set

We collected 17,424 individual records of dogs from Orange County Animal Services between February 2013 and February 2015 for data analysis. OCAS is a public, open-admission animal shelter and obtains its animals from owner surrenders and through the organization’s animal control duties within the county and surrounding municipalities. Records were collected via the shelter software program, Chameleon (HLP, Inc., USA), and the dogs’ intake date, intake type and subtype, outcome date and type, approximate age and primary breeds were used in our analysis.

Pit-bull-type breeds comprise 32% of dogs entering the Orange County shelter (5,592 out of 17,424). During the examined time period there was an overall increase in dog intake of approximately 3% (from 8,591 to 8,834), but that increase was spread evenly across pit bull and non-pit bull type dogs (*X*^*2*^ (1, *N* = 17,425) = 0.38, *p* = 0.54).

Outcome types were classified into three categories. “Adopt/Live Exit:” dogs that were adopted, returned to owner, placed in rescue or foster, transferred to another organization or relocated. “Return to owner:” dogs that came into the shelter but subsequently retrieved by their owner. “Euthanized/Died:” dogs that were euthanized at the shelter, were brought in for disposal after death or died at the shelter. Lastly, “Other:” dogs with all other outcomes or where no outcome was provided.

From their primary breed designations, dogs were placed into seven categories based on the American Kennel Club’s (New York, NY, USA) breed group system. The American Kennel Club classification system is based on the characteristics and historical functions of these breeds (http://www.akc.org). Breed categorizations used were Terrier, Hound, Working, Sporting, Herding, Non-Sporting and Toy. The Terrier group was further subdivided into Pit Bulls and Other Terriers (Table 3). Length of stay was calculated by subtracting the difference in days between intake and outcome dates.

**Table 3:** Breed Groups, Sub Groups & Associated Breed.

Breed Group	Associated Breeds
Terriers: Pit Bulls	Pit Bull, American Bulldog, American Pit Bull Terrier, American Staffordshire Terrier, Staffordshire Terrier, Bull Terrier, Miniature Bull Terrier
All Other Terriers	Jack Russell Terrier, Miniature Schnauzer, Rat Terrier, Cairn Terrier, Border Terrier, Norfolk Terrier, West Highland Terrier, Norwich Terrier, Scottish Terrier, Wire Fox Terrier, Manchester Terrier, Smooth Fox Terrier, Soft Coated Wheaten Terrier, Australian Terrier, Lakeland Terrier, Airedale Terrier, Skye Terrier, Irish Terrier, Welsh Terrier, Dandi Dinmont Terrier, Bedlington Terrier, Sealyham Terrier
Hound	Beagle, Dachshund (Standard), Catahoula Leopard Dog, Rhodesian Ridgeback, Dachshund (Miniature), Basset Hound, Dachshund (Long-Haired), Dachshund (Wire-Haired), Basenji, Treeing Walker Coonhound, Black & Tan Coonhound, American Foxhound, Greyhound, Bloodhound, Redbone Coonhound, Bluetick Coonhound, Whippet, Harrier, Otterhound, English Foxhound, Plott Hound, Grand Basset Griffon Vendeen, Treeing Tennessee Brindle, Redtick Coonhound, Petit Basset Griffon Vendeen, Pharaoh Hound, Irish Wolfhound, Afghan Hound
Working	Boxer, Rottweiler, Siberian Husky, Mastiff, Doberman Pinscher, Akita, Great Dane, Standard Schnauzer, Bullmastiff, Alaskan Malamute, Cane Corso, Great Pyrenees, Anatolian Shepherd, Neapolitan Mastiff, Saint Bernard (Smooth- Coated), Greater Swiss Mountain Dog, Presa Canario, Dogo Argentino, Giant Schnauzer, Portuguese Water Dog, Samoyed, Newfoundland, Saint Bernard (Rough-Coated), Dogue De Bordeaux
Sporting	Labrador Retriever, Cocker Spaniel, Golden Retriever, Pointer, Weimaraner, Vizsla, German Shorthaired Pointer, English Springer Spaniel, Treeing Cur, English Pointer, Brittany, Irish Setter, Flat-Coated Retriever, Wirehaired Pointing Griffon, Chesapeake Bay Retriever, English Setter, English Cocker Spaniel, Gordon Setter, Field Spaniel, Spinone Italiano, Clumber Spaniel, Nova Scotia Duck Tolling Retriever, Curly-Coated Retriever, German Wirehaired Pointer, Welsh Springer Spaniel
Herding	German Shepherd Dog, Border Collie, Australian Shepherd, Australian Cattle Dog, Smooth Collie, Cardigan Welsh Corgi, Pembroke Welsh Corgi, Shetland Sheepdog, Belgian Malinois, Rough Collie, Canaan Dog, Dutch Sheepdog, Australian Kelpie, Bearded Collie, Belgian Sheepdog
Non-Sporting	Poodle (Miniature), Chow Chow, Lhasa Apso, Boston Terrier, Chinese Shar-Pei, English Bulldog, Bichon Frise, Shiba Inu, Bulldog, Dalmatian, American Eskimo, Poodle (Standard), Schipperke, French Bulldog, Tibetan Terrier, Keeshond, Finnish Spitz, Tibetan Spaniel, Coton De Tulear, Lowchen, Jindo
Toy	Chihuahua (Short Coat), Shih Tzu, Yorkshire Terrier, Maltese, Pomeranian, Chihuahua (Long Coat), Miniature Pinscher, Pug, Pekingese, Poodle (Toy), Papillion, Silky Terrier, Brussels Griffon, Cavalier King Charles Spaniel, Chinese Crested, Japanese Chin, Italian Greyhound, Toy Fox Terrier, Affenpinscher, Havanese

#### Statistical Analyses

For the purposes of this analysis, completed canine records with outcomes from February 6, 2013 –February 5, 2014 prior to the labeling change were compared to those from February 6, 2014 –February 5, 2015. Expected versus actual frequency of adoption and euthanasia before and after the labeling change were analyzed for pit bulls and all other breed groups using chi-square tests. One-factor ANOVAs were utilized to assess change in length of stay before and after label removal for pit bulls and other breed groups, and post-hoc pairwise analyses were conducted using Dunnett’s T3 tests.

### Results and Discussion

Chi-square tests were performed to assess frequency of outcome types before and after the change in labeling practice. After breed labels were removed, adoptions of pit bulls were significantly higher than expected, *X*^*2*^ (2, *N* = 5,550) = 93.61, *p* < .001, while there was no significant difference from expected rates for all other terriers (X^2^ (2, *N =* 1,000) = 3.36, *p* = .19).

When breed was included on the kennel card, only 52% of entering pit bulls were adopted compared with 64% once the breed information was removed. This was mirrored by a 12% reduction in euthanasia of pit-bull-type dogs.

All other breed groups showed increases in adoption after the removal of cage card breed information. Working breeds showed an 8% increase in adoptions without breed on the kennel card (*X*^*2*^ (2, *N* = 1,205) = 9.24, *p* < .01) despite a 3.5% drop in the number of these breeds of dog entering the shelter. Boxers (+11%), Mastiffs (+15%), and Dobermans (+12%) accounted for the majority of the increase in adoptions (see Table 4 for adoption counts by breed group).

**Table 4:** Adoptions & All Live Exits By Breed Group With & Without Labels.

Breed Group	With Labels	Without Labels	Count Change
Terriers: Pit Bulls	1397	1813	416
All Other Terriers	350	399	49
Hound	406	425	19
Working	367	402	35
Sporting	858	845	13
Herding	383	439	56
Non-Sporting	337	411	74
Toy	1124	1169	45
Total	5222	5903	681

*Note*: Every Breed Group showed an increase in adoptions/live exits from year with labels (2013–2014) to year without labels (2014–2015).

In our analysis of length of stay, 56 outliers (values beyond 3 standard deviations above the maximum group mean) were removed from the analysis before a one-factor ANOVA was performed to analyze differences in length of stay for pit bulls before and after the change in labeling. Removal of breed labels significantly reduced pit bull length of stay, *F* (1,1742) = 24.37, *p* < .001. A one-way ANOVA was conducted to compare length of stay for all breeds after the change in labeling. The effect was significant, *F* (7,3762) = 29.86, *p* < .001 (see Table 5 for length of stay means and standard deviations by breed group). Post-hoc comparisons using Dunnett's T3 tests indicated differences between the mean length of stay for pit-bull-type dogs and all other breed groups before and after the change in labeling were significant (Smallest *t* = 5.01, largest = 12.90, df = 365–1818, each comparison at *p* < .001).

**Table 5:** Length of Stay of Adopted Dogs By Breed Group With & Without Labels.

Breed Group	With Labels		Without Labels		Change
	*M*	*SD*	*M*	*SD*	
Terriers: Pit Bulls	11.36	5.68	9.85	6.39	1.51
All Other Terriers	7.83	4.10	6.92	4.00	0.91
Hound	8.22	4.59	7.45	4.56	0.77
Working	9.08	4.76	7.30	5.58	1.78
Sporting	8.32	5.01	7.98	5.52	0.34
Herding	8.64	5.12	7.11	4.67	1.53
Non-Sporting	8.19	4.13	7.33	4.89	0.86
Toy	7.52	3.99	6.82	3.80	0.70
Total	8.80	4.98	8.03	5.38	0.77

*Note*: Every Breed Group showed a reduction in length of stay from year with labels (2013–2014) to year without labels (2014–2015).

With breed labels removed at OCAS, more pit bulls were adopted and their lengths of stay were reduced. While all other breed groups benefitted from these measures, the improvement in adoption rates for the pit-bull-type breeds was greater than for any other group. The one-and-a-half day reduction in length of stay for pit bulls was less than the reduction for Working and Herding groups, but nearly double the average decrease in length of stay observed shelter-wide. Pit bulls, however, continued to have the longest length of stay in the shelter relative to all other breed groups. The results of removing breed labels suggest that the pit bull breed labels were negatively altering adopter decision-making at OCAS.

## General Discussion

Our hypothesis that people would perceive the pit-bull-type dogs unfavorably relative to other breed types was supported by participants’ behavioral and adoptability ratings. Considering the total impact of the six rated characteristics assessed in Study 1, our findings support Wright et al.’s [30] assertion that morphological differences activate certain behavioral expectations about dogs regardless of information about the particular dog’s behavior. While stereotyping is often discussed in person perception, its definition as categorical assignment based on appearance and subsequent trait attribution [35,36] can also apply to perceptions about breeds of dogs [37].

Other breed stereotypes were also confirmed in our results. The Border Collie was rated significantly higher in perceived intelligence compared to the other dogs, paralleling the breed’s American Kennel Club standard where it’s described as “intelligent, keen, alert and responsive” (http://www.akc.org). Perceived adoptability was equally high for the Labrador Retriever and Border Collie but lower for the pit bull. In Protopopova et al. [10], adoption percentages by breed type also reflected these preferences. While lap breeds were preferred overall, herding and sporting breeds had adoption rates near 80% while fighting breeds (which included pit bulls) had rates below 50%.

In Study 1 we found that when participants viewed the pit bull with an elderly woman or male child, these handlers improved perceptions of the dog’s intelligence, friendliness and adoptability while lessening its perceived aggressiveness. Additionally, approachability increased with the elderly woman and difficulty to train declined with the child. The ability of these individuals to attenuate negative perceptions of the pit bull suggests that they could be serving as contextual primers whereby participants are passively influenced by their positive agency [38]. While previous studies have found evidence of trait contagion from dogs to owners [39], this is the first study to our knowledge that indicates a transference from handler to dog.

Breed has long been a focus of shelter research and is often implicated as an influencing factor in adoption success [14,15,40]. Our results in Study 2 point to a significant association between the pit bull label and length of stay, with pit-bull-type dogs waiting over three times as long to be adopted as their lookalike counterparts. Other studies have found similar associations of this group with negative outcomes. In Clevenger and Kass [19], pit bulls, Rottweilers and Chows were euthanized more often when compared to other breeds, and in Protopopova et al. [10] the fighting breed category (comprised 85% of pit bulls, Bulldogs, Boxers and Sharpeis) had the lowest adoption success and second longest length of stay.

In Study 3, we were surprised to find that, without labels, potential adopters viewed pit-bull-type dogs in videos as more attractive than their lookalikes. While lookalike breed labels were not found to have any impact on adopters’ perceptions compared to leaving dogs unlabeled, labels of pit bull breeds did reduce perceived attractiveness. Based on research into impression formation processes [41], it is likely that this negative impact occurred when reading the label and then accessing known attributes associated with the category of “pit bull.” The potency of the pit bull label suggests that this negatively perceived information had a stronger influence on potential adopters’ perceptions than more positive perceptions of lookalike breed labels [42].

New dog owners have indicated that one reason they ultimately acquired their dogs from a pet store, breeder or another non-adoption source is because the animal shelter did not have the type of dog they were looking for [43]. Perceived lack of certain breeds as portrayed on kennel cards and online profiles could be contributing to this attitude. Study 4 demonstrated that the removal of breed labels at Orange County Animal Services was associated with better adoption outcomes (increased adoptions and a shorter number of days awaiting adoption), not only for pit-bull-type dogs (although the benefit for this group was greatest), but for nearly all breeds.

Hoffman, Harrison, Wolff, and Westgarth [44] reported that shelter staff use physical appearance as the primary means of breed assignment for shelter dogs. Yet this method of breed identification has shown to be inaccurate when compared to DNA analysis [29,27,28]. The work of Scott and Fuller [45] shows that first- and second-generation crosses of two pure-breed dogs from different breeds evince a wide range of morphological diversity. This makes the premise of breed labeling at animal shelters, at best, inherently complex and, at worst, untenable. In Weiss, Miller, Mohan-Gibbons and Vela [9], researchers found appearance to be one of the most important reasons adopters chose their dogs, however their survey included breed as a component of appearance. Thus this study may also be implicating breed labels as a factor in adoption. Our findings here offer the possibility that by eliminating breed from adopter decision-making, preferred appearance may be disambiguated from perceptions about breed or breed-specific behaviors.

Despite the complex and multi-factorial nature of accurate labeling, it remains possible that breed designations made by animal shelters may be providing potential adopters with important information about the dog’s temperament not otherwise provided on the kennel card or by cursory inspection of the dog. At this time, we are uncertain how and to what degree behaviors emitted by dogs of unknown heritage influence decisions about their breed. Bollen and Horowitz [46] found that pit bulls and pit bull mixes showed the highest failure rates of any breed on the behavior evaluation used at a shelter to predict aggressiveness. Duffy, Hsu, and Serpell [25] reported that answers given by owners on the Canine Behavioral Assessment and Research Questionnaire (C-BARQ) yielded distinct differences between breeds in the prevalence and severity of aggression. Pit Bull terriers were found to be involved in incidents of aggression towards strangers only slightly more than average, but several epidemiological studies have found these dogs to be the most commonly implicated in injurious and fatal human bite cases [20,22–24]. Duffy et al. [25] did find that aggression directed towards unfamiliar dogs was significantly higher in pit-bull-type dogs compared to other dog breed groups.

Expectations about behavior associated with certain breeds may not be as consistent as was once thought. While reliable behavioral differences between dog breeds do exist, there is also a large amount of within-breed variation, which can be attributed to genetic and environmental causes as well as individual experiences [47]. How these types of influence converge in the behavior of individual mixed breed dogs is not well understood. When describing their studies of purebred and cross-bred dogs and the role of heredity in behavior, Scott and Fuller [45] wrote, “At the beginning of the hybridization experiment we were looking for genetic mechanisms to correspond with hypothetical traits. As data accumulated, it became clear that correlation between different tests given at different times and places were low—in other words, we found little evidence for pervasive traits affecting all aspects of behavior” (p. 323).

We conclude that breed labels in animal shelters are not providing adopters with the useful information they purport to, and removing them would be a relatively low-cost intervention that could improve adoptions and reduce lengths of stay for many—perhaps all—breed groups, including pit-bull-type dogs. However in an effort to fully understand the impact of breed label removal on adoption process, ongoing monitoring of return rates and bite-quarantine cases is recommended. As an alternative to breed designations on kennel cards, we suggest a better means of communicating the behavior of dogs in animal shelters would be through the use of a fully validated behavioral assessment [48]. While such measures are already utilized in many shelters [49,50], few, if any, of these behavioral assessments meet accepted criteria for standardization, validity and reliability for psychological testing [51,52]. Nonetheless, focusing research efforts on assessments that address these concerns would be beneficial to potential adopters and shelter dogs [53]. Follow-up studies comparing adopter satisfaction pre- and post-label removal at shelters would likely be useful in determining whether the absence of such breed information is detrimental to the adoption experience as well as if any other information is now perceived as more impactful in the decision-making process.

A limitation of Study 1 was in the presentation of the handler in photographs with the pit-bull-type dog. Unlike the work of Walsh et al. [5] where researchers used baseline photos of the dogs alone and digitally placed the person alongside, we created new images, which may have incorporated subtle differences in the dogs’ body language. This could have altered the perceptions of the participants, above and beyond the addition of the handlers. This methodological decision was made in an effort to create more life-like images of dogs and people interacting.

While multiple raters were utilized in determining matches between pit-bull-type dogs and lookalikes throughout our studies, it is possible that the photographs used in Study 2 were not representative of how the dogs appeared to adopters. In Study 3, we found that pit-bull-type dogs were seen more positively than lookalikes when the dogs were unlabeled. This suggests that despite our matching efforts, an interaction with kennel behavior may have occurred which influenced participants’ attractiveness scores. Protopopova et al. [34], who used the same videos as we have here (as well as others), found that certain behaviors, such as facing away from the front of the kennel, standing and moving back and forth, were associated with longer lengths of stay. In Study 3, carry-over effects between viewing the same videos twice (once with and once without labels) may be present; however because the orders of videos were randomly determined in both sets and displayed continuously, any effects that were present should influence participants’ ratings to an equal extent.

Conversations with shelter administration at Orange County Animal Services elucidated other changes in shelter practices, including additional advertising of adoptable pets and expanded operating hours, that were implemented after the removal of breed labels which likely contributed to improvements in adoption and length of stay outcomes in Study 4.

## Conclusions

We found that the pit-bull-type dog was perceived more negatively than the other breeds, but that impression was positively modulated by the presence of an elderly woman and a male child. Shelter length of stay for pit-bull-type dogs was longer than for lookalikes; however potential adopter perceptions did not differ when viewing these dogs in photographs without breed labels. In video recordings, perceptions of attractiveness were altered when dogs were labeled or unlabeled. Pit bull breed labels had a negative effect on the dogs’ perceived attractiveness, while lookalike labels did not have a positive impact on attractiveness compared to no label at all. In the shelter, removing breed labels was associated with increased adoptions and reduced length of stay for all breed groups, particularly pit-bull-type dogs. Given the inherent challenges of breed assignment based on morphology, removing breed labels from kennel cards and online adoption profiles may be a simple, low-cost strategy to improve shelter dog outcomes. With the limited usefulness of breed-specific information in describing the behavior of mixed breed dogs, a validated behavioral assessment would likely be a better way to inform potential adopters about the behavior of individual shelter dogs.

## Supporting Information

S1 Appendix Study 1 images. (PDF)

S2 Appendix Study 2 images. (PDF)

## References

[1] Coile DC. The dog breed bible. Hauppauge: Barron’s Educational Series; 2007.

[2] Serpell J. The domestic dog. Cambridge: Cambridge University Press; 1995.

[3] Notari L, Goodwin D. A survey of behavioural characteristics of purebred dogs in Italy. Appl Anim Behav Sci. 2007;103(1): 118–30.

[4] Bennett PC, Mornement K. Young adults’ familiarity with, and perceptions of, common dog breeds in Australia. J Vet Behav. 2009;4(2): 102.

[5] Walsh EA, McBride A, Bishop F, Leyvraz AM. Influence of breed, handler appearance and people’s experience of dogs on their perception of the temperament of a breed of dog in Ireland. Proceedings of the 16^th^ Annual Meeting of the International Society for Anthrozoology; 2007; Toyko, Japan.

[6] American Pet Products Association. U.S. pet-ownership estimates from the APPA for 2012. Available: http://www.humanesociety.org/issues/pet_overpopulation/facts/pet_ownership_statistics.html#.U0oh8uZdW_A. Accessed 30 January 2014.

[7] Wenstrup J, Dowidchuk A. Pet overpopulation: Data and measurement issues in shelters. J Appl Anim Welf Sci. 1999;2(4): 303–19. 16363935 10.1207/s15327604jaws0204_5

[8] American Society for the Prevention of Cruelty to Animals. FAQ, Pet statistics, 2012. Available: http://www.aspca.org/about-us/faq. Accessed 19 September 2014.

[9] Weiss E, Miller K, Mohan-Gibbons H, Vela C. Why did you choose this pet?: Adopters and pet selection preferences in five animal shelters in the United States. Animals. 2012;2(2): 144–59. 10.3390/ani2020144 26486914 PMC4494324

[10] Protopopova A, Gilmour AJ, Weiss RH, Shen JY, & Wynne CDL. The effects of social training and other factors on adoption success of shelter dogs. J Appl Anim Welf Sci. 2012;142(1): 61–8.

[11] Ramirez M. “My dog’s just like me”: Dog ownership as a gender display. Symb Interact. 2006;29(3): 373–91.

[12] Nemcova D, Novak P. Adoption of dogs in the Czech Republic. Acta Veterinaria Brno. 2003;72(3): 421–7.

[13] Lockwood R, Rindy K. Are “pit bulls” different? An analysis of the pit bull terrier controversy. Anthrozoos. 1997;1: 2–8.

[14] Posage JM, Bartlett PC, Thomas DK. Determining factors for successful adoption of dogs from an animal shelter. J Am Vet Med Assoc. 1996;213(4): 478–82. 9713528

[15] Lepper M, Kass PH, Hart LA. Prediction of adoption versus euthanasia among dogs and cats in a California animal shelter. J Appl Anim Welf Sci. 2002;5(1): 29–42. 12738587 10.1207/S15327604JAWS0501_3

[16] Dowling-Guyer S, Marder A, D’Arpino S. Behavioral traits detected in shelter dogs by a behavior evaluation. Appl Anim Behav Sci. 2011;130(3): 107–114.

[17] Brown WP, Davidson JP, Zuefle ME. Effects of phenotypic characteristics on the length of stay of dogs at two no kill animal shelters. J Appl Anim Welf Sci. 2013;16(1): 2–18. 10.1080/10888705.2013.740967 23282290

[18] DeLeeuw, JL. Animal shelter dogs: Factors predicting adoption versus euthanasia Doctoral dissertation, Wichita State University. 2010. Available: http://soar.wichita.edu/bitstream/handle/10057/3647/d10022_DeLeeuw.pdf?sequence=1

[19] Clevenger J, Kass PH. Determinants of adoption and euthanasia of shelter dogs spayed or neutered in the University of California veterinary student surgery program compared to other shelter dogs. J Vet Med Educ. 2003;30(4): 372–378. 14976625 10.3138/jvme.30.4.372

[20] Sacks JJ, Sattin RW, Bonzo SE. Dog bite-related fatalities from 1979 through 1988. J Am Vet Med Assoc. 1989;262(11): 1489–1492. 10.1001/jama.262.11.1489 2769900

[21] Sacks JJ, Lockwood R, Hornreicht J, Sattin RW. Fatal dog attacks, 1989–1994. Pediatrics. 1996;97(6): 891–895. 8657532

[22] Sacks JJ, Sinclair L, Gilchrist J, Golab GC, Lockwood R. Breeds of dogs involved in fatal human attacks in the United States between 1979 and 1998. J Am Vet Med Assoc. 2000;217(6): 836–840. 10997153 10.2460/javma.2000.217.836

[23] Kaye AE, Belz JM, Kirschner RE. Pediatric dog bite injuries: A 5 year review of the experience at the Children’s Hospital of Philadelphia. Plast Reconstr Surg. 2009;124(2): 551–558. 10.1097/PRS.0b013e3181addad9 19644273

[24] O'Brien DC, Andre TB, Robinson AD, Squires LD, Tollefson TT. Dog bites of the head and neck: an evaluation of a common pediatric trauma and associated treatment. Am J Otolaryngol. 2015;36(1): 32–38. 10.1016/j.amjoto.2014.09.001 25311183 PMC4261032

[25] Duffy DL, Hsu Y, Serpell JA. Breed differences in canine aggression. Appl Anim Behav Sci. 2008;114(3): 441–460.

[26] Patronek GJ, Sacks JJ, Delise KM, Cleary DV, Marder AR. Co-occurrence of potentially preventable factors in 256 dog bite–related fatalities in The United States (2000–2009). J Am Vet Med Assoc. 2013;243(12): 1726–1736. 10.2460/javma.243.12.1726 24299544

[27] Voith V, Ingram E, Mitsouras K, Irizarry K. Comparison of adoption agency breed identification and DNA breed identification of dogs. J Appl Anim Welf Sci. 2009;12(3): 253–262. 10.1080/10888700902956151 20183478

[28] Voith VL, Trevejo R, Dowling-Guyer S, Chadik C, Marder A, Johnson V et al Comparison of visual and DNA breed identification of dogs and inter-observer reliability. Am J Sociol Res, 2013;3(2): 1729.

[29] Olson, KR, Levy, JK, Norby, B, Broadhurst, JE, Jacks, S, Reeves, RC et al. Pit bull-type dog identification in animal shelters. Fourth Annual Maddie’s Shelter Medicine Conference. 2011.

[30] Wright JC, Smith A, Daniel K, Adkins K. Dog breed stereotype and exposure to negative behavior: Effects on perceptions of adoptability. J Appl Anim Welf Sci. 2007;10(3): 255–265. 17645409 10.1080/10888700701353956

[31] Patronek GJ, Glickman LT, Moyer MR. Population dynamics and the risk of euthanasia for dogs in an animal shelter. Anthrozoos. 1995;8(1): 31–43.

[32] Salman MD, New JG Jr, Scarlett JM, Kass PH, Ruch-Gallie R, Hetts S. Human and animal factors related to relinquishment of dogs and cats in 12 selected animal shelters in the United States. J Appl Anim Welf Sci. 1998;1(3): 207–226. 16363966 10.1207/s15327604jaws0103_2

[33] Cohen J. Statistical power analysis for the behavioral sciences. Hillsdale: L Erlbaum Associates; 1988.

[34] Protopopova A, Mehrkam LR, Boggess MM, & Wynne CDL. In-kennel behavior predicts length of stay in shelter dogs. PloS One. 2014;9(12): e114319 10.1371/journal.pone.0114319 25551460 PMC4281133

[35] Allport GW. The nature of prejudice. Cambridge: Addison-Wesley; 1954.

[36] Tajfel H. Cognitive aspects of prejudice. J Soc Issues. 1969; 25: 79–97. 10.1017/s0021932000023336 5373848

[37] Kwan VS, Gosling SD, John OP. Anthropomorphism as a special case of social perception: A cross-species social relations model analysis of humans and dogs. Soc Cogn. 2008; 26(2): 129–142.

[38] Bargh JA, Chen M, Burrows L. Automaticity of social behavior: Direct effects of trait construct and stereotype activation on action. J Pers Soc Psychol. 1996; 71(2): 230–244. 8765481 10.1037//0022-3514.71.2.230

[39] Mae L, McMorris LE, Hendry JL. Spontaneous trait transference from dogs to owners. Anthrozoos. 2004;17: 225–243.

[40] Diesel G, Smith H, Pfeiffer DU. Factors affecting time to adoption of dogs rehomed by a charity in the UK. Anim Welf. 2007;16(3): 353–360.

[41] Fiske ST, Neuberg SL. A continuum of impression formation, from category-based to individuating processes: Influences of information and motivation on attention and interpretation. Adv Exp Soc Psychol. 1990;23: 1–74.

[42] Ito TA, Larsen JT, Smith NK, Cacioppo JT. Negative information weighs more heavily on the brain: The negativity bias in evaluative categorizations. J Pers Soc Psychol. 1998;75(4): 887 9825526 10.1037//0022-3514.75.4.887

[43] Maddalena, SD, Zeidman, S, Campbell, K. An empirical look at public perceptions and attitudes about pet adoption and spay/neuter. Society of Animal Welfare Administrators Conference. 2012.

[44] Hoffman CL, Harrison N, Wolff L, Westgarth C. Is that dog a pit bull? A cross-country comparison of perceptions of shelter workers regarding breed identification. J Appl Anim Welf Sci. 2014;17(4): 322–339. 10.1080/10888705.2014.895904 24673506 PMC4160292

[45] Scott JP, & Fuller JL. Genetics and the social behavior of the dog. Chicago: University of Chicago Press; 1965.

[46] Bollen KS, Horowitz J. Behavioral evaluation and demographic information in the assessment of aggressiveness in shelter dogs. Appl Anim Behav Sci. 2008;112(1): 120–135.

[47] Mehrkam LR, & Wynne CD. Behavioral differences among breeds of domestic dogs (Canis lupus familiaris): Current status of the science. Appl Anim Behav Sci. 2014;155: 12–27.

[48] Taylor KD, Mills DS. The development and assessment of temperament tests for adult companion dogs. J Vet Behav. 2006;1(3): 94–108.

[49] Mornement KM, Coleman GJ, Toukhsati S, Bennett PC. A review of behavioral assessment protocols used by Australian animal shelters to determine the adoption suitability of dogs. J Appl Anim Welf Sci. 2010;13(4): 314–329. 10.1080/10888705.2010.483856 20865615

[50] King T, Marston LC, Bennett PC. Breeding dogs for beauty and behaviour: Why scientists need to do more to develop valid and reliable behaviour assessments for dogs kept as companions. Appl Anim Behav Sci. 2012;137(1): 1–12.

[51] Haverbeke A, Pluijmakers J, Diederich C. Behavioral evaluations of shelter dogs: Literature review, perspectives, and follow-up within the European member states's legislation with emphasis on the Belgian situation. J Vet Behav. 2015; 10(1): 5–11.

[52] Rayment DJ, De Groef B, Petersx RA, Marston LC. Applied personality assessment in domestic dogs: Limitations and caveats. Appl Anim Behav Sci. 2015;163: 1–18.

[53] Overall KL. The mismeasure of behavior: Identifying tests meaningful to the species studied. J Vet Behav. 2015; 10(1): 1–4.

