# Supplementary material for: What’s in a Name? Effect of Breed Perceptions & Labeling on Attractiveness, Adoptions & Length of Stay for Pit-Bull-Type Dogs
Source: PLoS One. 2016 Mar 23;11(3):e0146857. doi: 10.1371/journal.pone.0146857 (PMC4805246; doi:10.1371/journal.pone.0146857)
Supplement: S1 Appendix — (PDF) [file pone.0146857.s001.pdf]

Supporting Information

S1 Appendix A

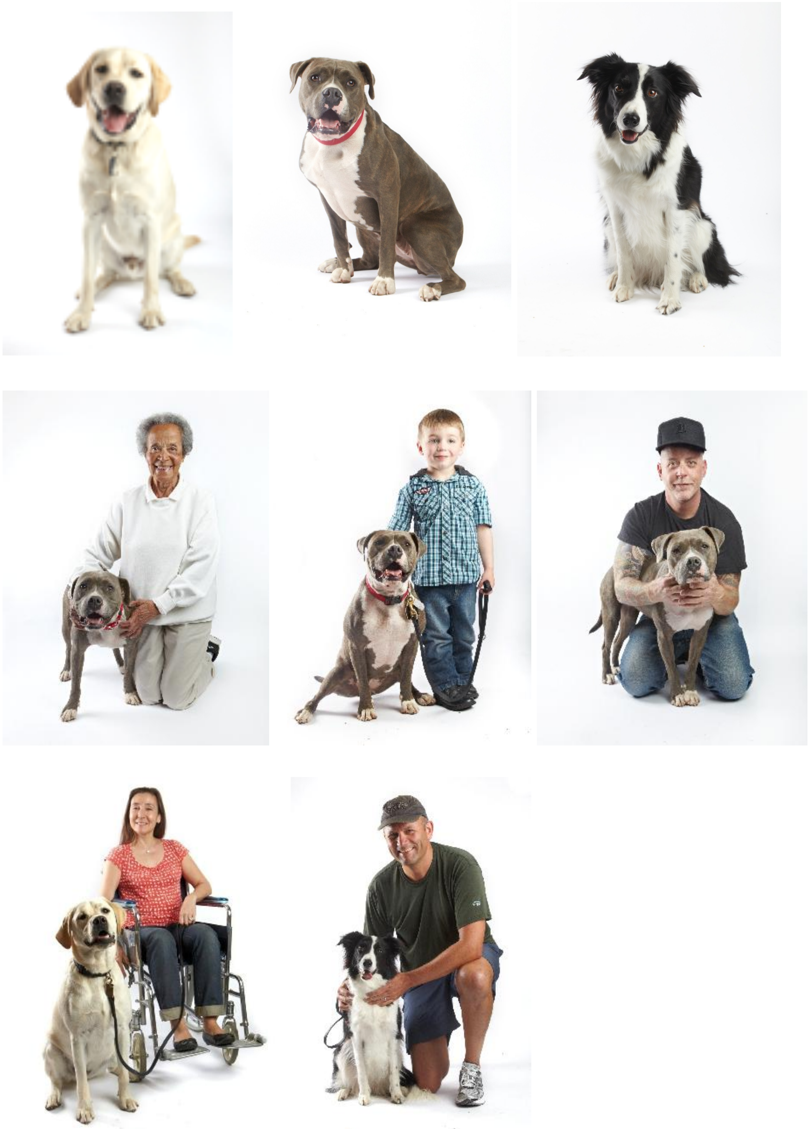

S1 Appendix A. Images used in Study 1 with three dog breed exemplars, five dog and handler images – including three pit bull handler conditions with the elderly woman, male child and rough adult male.
