# Supplementary material for: What’s in a Name? Effect of Breed Perceptions & Labeling on Attractiveness, Adoptions & Length of Stay for Pit-Bull-Type Dogs
Source: PLoS One. 2016 Mar 23;11(3):e0146857. doi: 10.1371/journal.pone.0146857 (PMC4805246; doi:10.1371/journal.pone.0146857)
Supplement: S2 Appendix — (PDF) [file pone.0146857.s002.pdf]

Supporting Information

S2 Appendix B

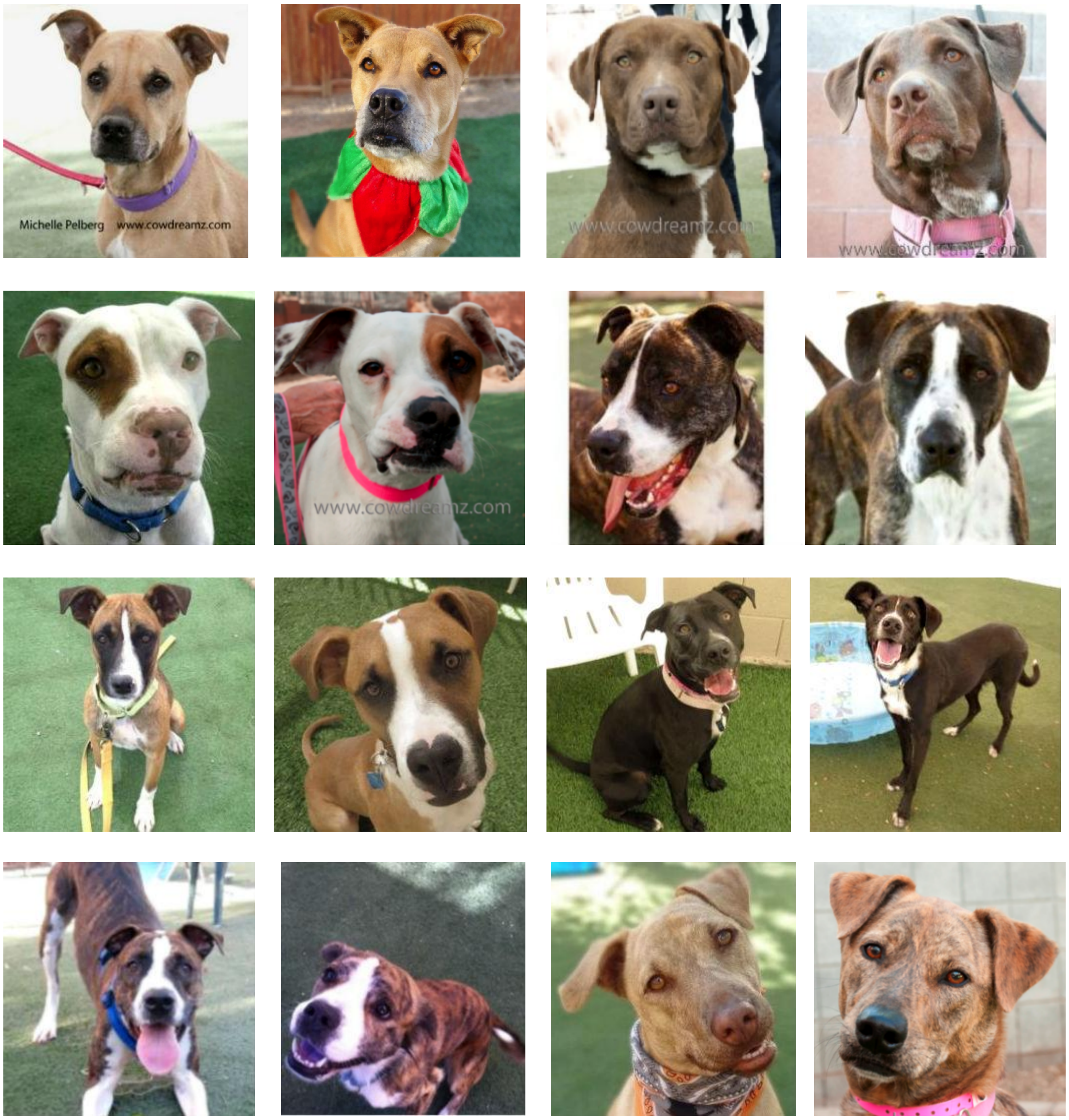
Michelle Pelberg www.cowdreamz.com
www.cowdreamz.com
www.cowdreamz.com

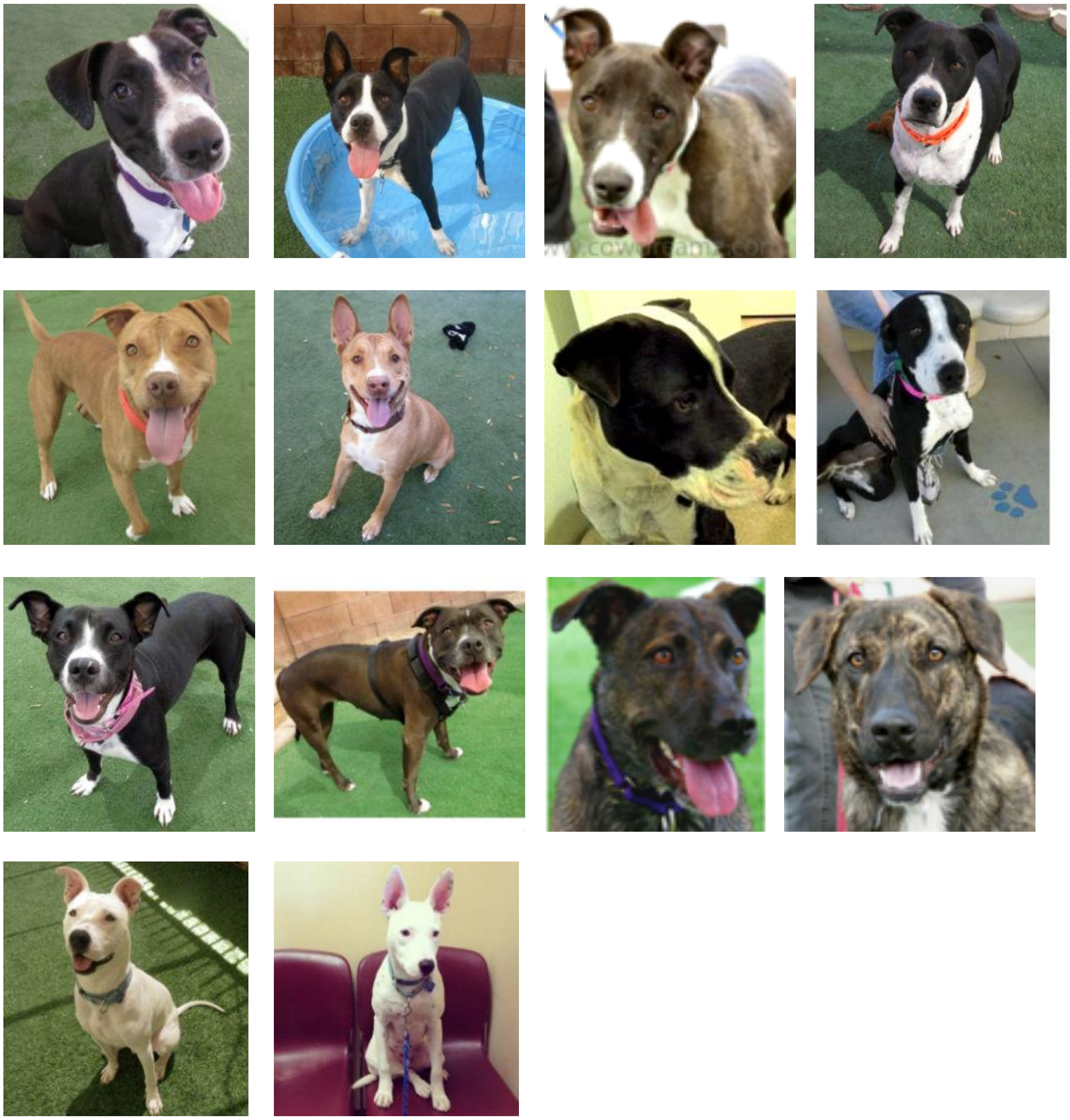

S2 Appendix B. Images used in Study 2. Images were randomized but are displayed here in matching pairs with dogs labeled as pit-bull-type breeds on the left and lookalike dogs of different breeds on the right.
